# The Dark Side of the Force – Constraints and Complications of Cell Therapies for Stroke

**DOI:** 10.3389/fneur.2015.00155

**Published:** 2015-07-20

**Authors:** Johannes Boltze, Antje Arnold, Piotr Walczak, Jukka Jolkkonen, Lili Cui, Daniel-Christoph Wagner

**Affiliations:** ^1^Department of Cell Therapy, Fraunhofer-Institute for Cell Therapy and Immunology, Leipzig, Germany; ^2^Translational Center for Regenerative Medicine, University of Leipzig, Leipzig, Germany; ^3^Division of MR Research, Russell H. Morgan Department of Radiology and Radiological Science, Johns Hopkins University School of Medicine, Baltimore, MD, USA; ^4^Institute for Cell Engineering, Johns Hopkins University, Baltimore, MD, USA; ^5^Department of Neurology, Institute of Clinical Medicine, University of Eastern Finland, Kuopio, Finland

**Keywords:** ischemic stroke, cell therapy, cell transplantation, translational research, clinical trial, side effect, complication, safety

## Abstract

Cell therapies are increasingly recognized as a promising option to augment the limited therapeutic arsenal available to fight ischemic stroke. During the last two decades, cumulating preclinical evidence has indicated a substantial efficacy for most cell treatment paradigms and first clinical trials are currently underway to assess safety and feasibility in patients. However, the strong and still unmet demand for novel stroke treatment options and exciting findings reported from experimental studies may have drawn our attention away from potential side effects related to cell therapies and the ways by which they are commonly applied. This review summarizes common and less frequent adverse events that have been discovered in preclinical and clinical investigations assessing cell therapies for stroke. Such adverse events range from immunological and neoplastic complications over seizures to cell clotting and cell-induced embolism. It also describes potential complications of clinically applicable administration procedures, detrimental interactions between therapeutic cells, and the pathophysiological environment that they are placed into, as well as problems related to cell manufacturing. Virtually each therapeutic intervention comes at a certain risk for complications. Side effects do therefore not generally compromise the value of cell treatments for stroke, but underestimating such complications might severely limit therapeutic safety and efficacy of cell treatment protocols currently under development. On the other hand, a better understanding will provide opportunities to further improve existing therapeutic strategies and might help to define those circumstances, under which an optimal effect can be realized. Hence, the review eventually discusses strategies and recommendations allowing us to prevent or at least balance potential complications in order to ensure the maximum therapeutic benefit at minimum risk for stroke patients.

## Introduction

Therapeutic stem cell research represents one of the most vibrant fields in regenerative medicine. Embryonic, fetal, and adult stem cells are believed to exert multiple therapeutic actions. These range from potential tissue regeneration over the support of local endogenous repair attempts to the beneficial modulation of systemic immune responses. The still ongoing discovery of this tremendous therapeutic potential has fueled the imagination of researchers and clinicians to develop novel therapeutic strategies and to treat disorders, which have been considered untreatable for decades. Among those, ischemic stroke plays a primary role. Stroke is a worldwide predominant cause of death and acquired disability in adulthood (1). The only currently available treatment is thrombolysis, being restricted by a narrow time window (2) and a number of contraindications (3). Together, these limitations exclude the majority of patients from successful and causal treatment. On the other hand, numerous scientific reports corroborated the therapeutic benefit provided by stem cell populations in stroke. This is exemplified by the improvement of neurofunctional deficits (4), reduction of infarct volume, an extension of the time windows for intervention (5, 6), pro-regenerative cerebral reorganization (7), and potentially even limited tissue restoration (8), as well as mitigation of post-stroke neuroinflammation (9). Consequently, first early stage clinical studies are underway to confirm safety and to collect evidence for the therapeutic benefit of stem cell-based treatments in human stroke patients (10).

However, the well-founded enthusiasm for cell therapies and the urgent need for novel therapeutic approaches seem to have drawn our attention away from possible complications of stem cell applications in stroke. Since each therapeutic intervention comes at the risk of undesirable side effects, such side effects would not generally compromise the overall value of stem cell therapies. They could, however, significantly limit the safety, efficacy, as well as successful translation of stem cell-based experimental treatment concepts into clinically available therapies. We therefore argue that potential side effects deserve a closer and more thorough look. Moreover, some side effects might be specific to stroke because important pathophysiological aspects such as blood brain barrier (BBB) breakdown, perilesional hyperexcitability, systemic immunodepression and others differ from those in the other central nervous system (CNS) pathologies. This review summarizes current preclinical and clinical evidence for risks arising from therapeutic use of stem cell populations and the means by which the therapies are commonly applied. It also describes major translational hurdles, which arise from undesirable interactions between the cell transplant and its local pathophysiological environment.

## Stem Cell Populations for Stroke Treatment: An Overview

A broad variety of embryonic, fetal, and adult stem cells (including mixed, stem cell-containing populations) have been investigated regarding their therapeutic potential in stroke. The effects exerted by the cells can in principle be discriminated into two main classes: cell replacement and stimulation of endogenous recovery and repair. A restorative capacity has been assumed for more naïve, pluri-, and multipotent stem cell populations, characterized by a distinct proliferation and differentiation potential. While this potential theoretically enables the cells to directly repair and replace damaged brain tissue after stroke, it is currently unclear whether this has a significant impact in practice (11). Next to a possible restorative potential, most stem cell populations are also believed to support endogenous repair processes indirectly by the secretion of cytokines, growth factors, and other messenger molecules. These can beneficially modulate endogenous reorganization and response processes following stroke and are assumed to represent the primary mode of action for adult stem cell populations. Being commonly summarized as so-called “bystander effects,” these processes can also exert a systemic impact on the host organism.

Table 1 summarizes the most relevant cell populations being currently under investigation as stroke therapeutics. Since several hundred preclinical studies have been published so far, the overview can only provide exemplary or key references. Table 1 also indicates whether first clinical experience with the respective cell populations has been collected using a scientific approach. This includes company driven, registered clinical trials, but excludes commercial single case treatments as occasionally offered by private clinics.

**Table 1:** **Overview on cell populations being investigated for stroke therapy**.

Cell population	Cell source	Key reference	Cell diameter (volume)	Therapeutic effects	Transplantation paradigms		Adverse events reported	Clinical trial
					Modality	Route	
**EMBRYONIC STEM CELLS (ESCs)**								
Murine ESC	Blastocysts	(12, 13)	8 μm (270 μm^3^)	Neuronal and glial differentiation	Allogeneic, xenogeneic	Intraparenchymal	Tumor formation (higher following allogeneic transplantation)	No
							Tissue overgrowth	
							Immunological response	

Embryonic NSCs	Derived from murine ESCs	(12, 14)	Not investigated	Improved functional recovery	Allogeneic, xenogeneic	Intraparenchymal	Tumor formation reported	No
	Derived from human ESCs	(15, 16)		Neuronal and glial differentiation, integration			Tissue overgrowth and secondary host tissue injury	
	Derived from monkey ESCs	(17)		Glial scar reduction/modulation			Immunological responses	
				Lesion size reduction			
**FETAL STEM CELLS**								
NSCs	Human fetal brain specimen (usually 1st trimester)	(7, 18)	16 μm (2150 μm^3^)	Improved functional recovery	Xenogeneic	Intraparenchymal, intravenous	Strong immunological response	Yes
				Enhanced neuroplasticity			Tissue overgrowth	
				Lesion size reduction Neuronal and glial differentiation Anti-apoptosis/neuroprotection			Tumor/neoplasm formation	
							Ectopic engraftment and tissue overgrowth	
**ADULT STEM CELLS**								
Neural precursor cells	Subventricular zone (rodents)	(19, 20)	16 μm (2150 μm^3^)	Improved functional recovery	Allogeneic syngeneic	Intraparenchymal, intravenous	Strong host immunological responses Tissue overgrowth	Yes
				Neuroprotection				
				Glial scar reduction/modulation	
				Enhanced endogenous neurogenesis	
				Glial scar reduction/modulation	
				Anti-inflammation	

Human multipotent adult progenitor cells (MAPC^®^)	Bone marrow	(21)	15–18 μm (1750–2150 μm^3^)	Anti-apoptosis	Xenogeneic, allogeneic	Intravenous	Non-reported so far, but immunological responses, clotting phenomena/microembolism (although to a smaller extent than MSCs) cannot be excluded after systemic administration	Yes
				Angiogenesis	
				Anti-inflammation	
				Glial scar reduction/modulation	
				Enhanced endogenous neurogenesis	

Dental pulp multipotent stem cells	Dental pulp	(22)	Not investigated	Improved functional recovery Neuronal differentiation abilities	Xenogeneic	Intraparenchymal	Non-reported so far, but at least immunological responses may not be excluded after systemic administration	No

HUCB-NSC	Cord blood	(23)	15 μm (1750 μm^3^)	Neuronal and astroglial differentiation Improved functional recovery (?)	Xenogeneic	Intraparenchymal	Strong immunological responses, only partially preventable by immunosuppression	No

MSCs	Bone marrow	(21, 24–26)	18 μm (3050 μm^3^)	Improved functional recovery	Autologous, allogeneic, xenogeneic	Intraparenchymal, intraarterial, intravenous, intrathecal, intranasal	Microembolism	Yes
	Cord blood Placenta Adipose tissue	(27)		Neuronal and glial differentiation (?) Anti-inflammation/immunomodulation Anti-apoptosis Angiogenesis Neuroprotection Glial scar reduction/modulation			Increased mortality in diabetic animals Neointima formation in the internal carotid artery (predominantly under diabetic conditions) Enhanced atherosclerosis (predominantly under diabetic conditions)	
		(28)		Enhanced endogenous neurogenesis				
		(29)						

Hematopoietic stem/progenitor cells	BM-derived hematopoietic stem cells	(30, 31)	6–10 μm (115–520 μm^3^)	Improved functional recovery Neuronal and glial differentiation (?) Anti-inflammation/immunomodulation	Allogeneic syngeneic, xenogeneic	Intraparenchymal, intravenous	Generally not well investigated, but probably comprising: immunological responses and GvHD	Yes
	Peripheral blood	(32)			
	Cord blood	(8, 33)			
				Neuroprotection	
				Enhanced neuroplasticity	
				Enhanced endogenous neurogenesis	
				Angiogenesis	

MNC	BM	(34)	7 μm (180 μm^3^)	Improved functional recovery	Syngeneic, allogeneic, xenogeneic	Intraparenchymal, intraarterial, intravenous	Immunological responses	Yes
	Cord blood	(5, 33)		Neuronal and glial differentiation (?)	
	Peripheral blood	(35)		Neuroprotection	
				Anti-inflammation/immunomodulation	
				Lesion size reduction	
**BIOENGINEERED (ARTIFICIAL) STEM CELL POPULATIONS**								
iPS cells	Diverse, often (foreskin), or embryonic fibroblasts	(36, 37)	Not investigated, probably similar to ESCs	Improved functional recovery	Allogeneic, xenogeneic	Intraparenchymal	Teratoma formation (particularly after stroke) and tissue overgrowth	No
				Neuronal and glial differentiation	
				Neuroprotection	
				Lesion size reduction	
				Anti-inflammation/immunomodulation	

iN cells	iPS-cell-derived neural stem cells	(38, 39)	Not investigated	Neuronal and astroglial differentiation	Xenogeneic	Intraparenchymal	Teratoma formation (?)	No
				Improved functional recovery (?)	
				Lesion size reduction	

**Figure 1:**
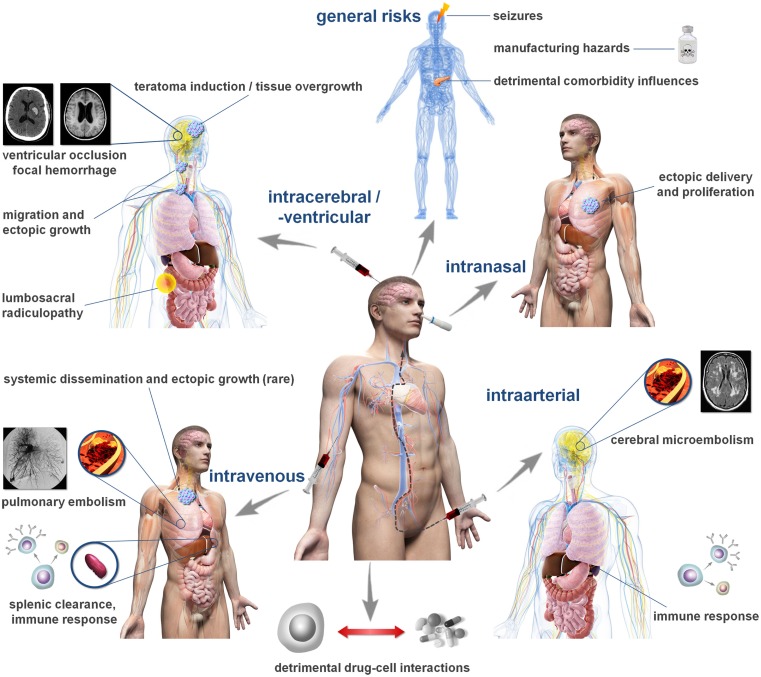
**Cell administration routes and related complications**. The figure depicts common routes investigated for cell and stem cell transplantation following stroke and potentially associated complications. The frequency of such complications can hardly be estimated from the available data in humans and may vary significantly between the individual elements. Please note that not all cell populations exhibit the same risk profile. For details, please consult Tables 1 and 2.

Thus far, over 20 clinical studies have been published and more than 20 phase I/II trials are ongoing (40). Several cell products have been investigated, with mesenchymal stem cells (MSCs), being the most common population applied. The intravenous delivery route is used most often with a typical dose range from 1 × 10^6^ to 1 × 10^7^ cells/kg bodyweight. Each clinical trial is preceded by a safety assessment approved by the responsible regulatory body. Different national regulations exist, but requirements are commonly less strict for early stage (phase I/II) clinical trials and routinely rely on standard *in vivo* safety investigations and aspects of cell manufacturing. Most studies do not report adverse events apart from minor and unspecific ones including transient fever, nausea, skin itching, or appetite loss (41), but more serious adverse events have also been reported. While trends toward favorable outcomes are reported, they must be interpreted cautiously as early stage clinical trials are neither designed nor powered to reliably detect efficacy. The detection of less frequent, potentially more severe adverse events may likewise be masked by the relatively low-statistical power of current early stage clinical trials, restricting the occurrence of such events to a small number of individual cases. Moreover, these trials often lack appropriate control groups, which would allow a firm conclusion on potential side effects. This assumption is supported by the increasing body of evidence for potential cell therapy side effects from preclinical investigation. Table 2 summarizes current clinical indications for complications related to cell therapy. The Figure 1 illustrates potential detrimental effects of cell therapies in relation to the selected route of cell administration.

**Table 2:** **Current clinical trials investigating cell therapies for stroke including reported complications**.

Study	Design	Patients	Cell type	Cell source	Transplantation procedure				Reported adverse events
		Treated/controls			Time window	Route	*n*	Cell number/dose and further details	
**COMPLETED CLINICAL TRIALS**									
(42)	Observer-blinded, phase I	12/0	Predifferentiated neuronal cells	Allogeneic NT2/D1 precursors	0.5–6 years	Intraparenchymal	1×	4 Patients: single trajectory (2 × 10^6^/3 deposits)	None study-related reported
								8 Patients: randomized either (2 × 10^6^/3 deposits) or three trajectories (3 × 2 × 10^6^ cells in 3 × 3 deposits)	
(43)	Randomized, observer-blinded, phase II	14/4	Predifferentiated neuronal cells	Allogeneic NT2/D1 precursors	1–6 years	Intraparenchymal	1×	7 Patients: 5 × 10^6^ cells in 25 deposits	Single seizure, syncopic episode, subdural hematoma
								7 Patients: 10 × 10^6^ cells in 25 deposits	No cell-related complication
(44)	Randomized, observer-blinded, phase I/II	5/25	MSC	Autologous bone marrow	5 weeks	Intravenous	2×	5 × 10^7^ each (at 5 and 7 weeks)	None reported
(45)	Open-label, phase I	5/0	Fetal lateral ganglionic eminence cells	Xenogeneic (porcine)	1.5–10 years	Intraparenchymal	1×	4 Patients: 1 × 10^7^ cells per trajectory (up to 5 each)	Temporary worsening of motor deficits, seizures
								1 Patient: 8 × 10^8^ cells in one trajectory	Trial terminated due to side effects in 2 patients
(46)	Open-label, phase I	5/0	MNC	Autologous bone marrow	1–10 years	Intraparenchymal	1×	1.4–5.5 × 10^7^ in 6–15 trajectories and 46–88 deposits	Headache, drowsiness, nausea, blood pressure increase, hyperglycemia, fever, dysesthesia
(47)	Open-label, phase I	6/0	MNC	Autologous bone marrow	<90 days	Intraarterial	1×	1.25–5 × 10^8^	None study-related reported
(48)	Observer-blinded, phase II	16/36	MSC	Autologous bone marrow	5 weeks	Intravenous	2×	5 × 10^7^ each (at 5 and 7 weeks)	None study-related reported
(49)	Open-label, phase I	6/0	MNC	Autologous bone marrow	2–3 months	Intraarterial	1×	1–5 × 10^8^	Generalized seizure (2 of 6 patients)
(50)	Open-label, non-randomized, phase I/II	6/6	MSC	Autologous bone marrow	3–12 months	Intravenous	1×	5–6 × 10^7^	None study-related reported
(41)	Open-label, phase I	12/0	MSC	Autologous bone marrow	36–133 days	Intravenous	1×	0.6–1.6 × 10^8^	Mild fever, nausea, appetite loss, skin itching
(51)	Open-label, phase I	10/0	MNC	Autologous bone marrow	24–72 h	Intravenous	1×	7–10 × 10^6^ cells/kg	None study-related reported
(52)	Open-label, phase I	20/0	MNC	Autologous bone marrow	3–7 days	Intraarterial	1×	22 × 10^6^	None study-related reported
(53)	Observer-blinded, phase I/II	10/10	MNC	Autologous bone marrow	5–9 days	Intraarterial	1×	1.6 × 10^8^ (average)	Seizures (2 of 10 patients)
(54)	Open-label, phase I	11/0	MNC	Autologous bone marrow	7–30 days	Intravenous	1×	1.9–185 × 10^6^ (average: 8.0 × 10^6^)	One re-infarction (etiology not clear)
(50)	Non-randomized, phase I/II	20 (6 versus 14)/20	MSC (*n* = 6), MNC (*n* = 14)	Autologous bone marrow	0.25–2 years	Intravenous	1×	5–6 × 10^7^	None study-related reported
(55)	Open-label, phase I	4/0	MSC	Allogeneic umbilical cord	0.5–2 months	Intraarterial		2 × 10^7^	None reported
(56)	Observer-blinded, phase I/II (hemorrhagic stroke)	60/40	MNC	Autologous bone marrow	5–7 days	Lesion cavity	1×	2.5–22.2 × 10^6^ (in 3.5 ml)	Fever, chest pain in 1 case, unspecified pulmonary tumor in 1 case
(57)	Open-label, non-randomized, phase I	12 (5 versus 7)/0	MNC	Autologous bone marrow	19–89 days	Intravenous (*n* = 5)	1×	1–5 × 10^8^	Seizures in 5 of 5 patients in intravenous group
						Intraarterial (*n* = 7)			Seizures in 2 of 7 patients in intraarterial group
									Neurological worsening after seizures in 1 case
(58)	Open-label, phase I	8/0	CD34^+^	Autologous bone marrow	1–7 years	Intrathecal	4–5×	0.8–3.3 × 10^7^ (per injection, 1 week interval)	None reported
(59)	Open-label, non-randomized, phase I	5/0	CD34^+^	Autologous bone marrow	<7 days	Intraarterial	1×	<1.0 × 10^8^	Non-study-related reported
(60)	Single-blinded, randomized phase II	15/15	CD34^+^	Autologous peripheral blood	0.6–5 years	Intraparenchymal	1×	3–8 × 10^6^	None reported
(61)	Blinded, randomized, phase II	85/35	MNC	Autologous bone marrow	18.5 days (mean)	Intravenous	1×	2.8 × 10^8^	None reported
(62)	Open-label, phase I/II	24/0	MNC	Autologous bone marrow	40.5 months (mean)	Intrathecal	1×	1 × 10^6^/kg	None reported
(63) (Public presentation)	Randomized, double blind, phase II	140 Targeted 65/61 Finally evaluated	Multipotent adult progenitor cells (MAPC^®^)	Adult bone marrow	24–48 h	Intravenous	1×	Dose selection in 8/8 patients: high (1.2 × 10^9^) and low dose (4 × 10^8^) paradigms, all evaluated patients in the cell therapy group received high-dose treatment	Overall lower frequency of life-threatening events and less pulmonary complications in cell-treated patients
**PUBLISHED SINGLE CASES**									
(64)	Individual treatment attempt (ataxia telangiectasia)	1/0	Neural stem cells	Allogeneic fetal tissue	“Chronic”	Intraparenchymal and CSF space	N/A	“Multiple injections”	Local ectopic tissue overgrowth/teratoma formation requiring surgical intervention
(65)	Individual treatment attempt (global hypoxia)	1/0	Neural progenitors	Autologous cord blood	6 months	Intrathecal (ventricular)	1×	12 × 10^6^ cells/0.5 ml	Fever
(66)	Individual treatment attempt	1/0	Neural stem cells	Unclear, probably allogeneic	“Chronic”	Intrathecal	N/A	“Multiple injections”	Severe inflammatory polyradiculopathy
**ONGOING CLINICAL TRIALS**									
NCT01678534	Randomized, controlled, double blind, phase II	20	MSC	Allogenic adipose tissue	2 weeks	Intravenous	1×	1 × 10^6^/kg	Ongoing
NCT01151124	Open-label, phase I	12	Neural stem/progenitor cells (CTX)	Allogeneic (human cortical neuroepithelium)	0.5–5 years	Intraparenchymal	1×	Dose escalation: 2×, 5×, 10×, 20 × 10^6^	Ongoing
NCT02117635	Open-label, phase II	up to 62 (two-stage)	Neural stem/progenitor cells (CTX)	Allogeneic (human cortical neuroepithelium)	4 weeks	Intraparenchymal	1×	2 × 10^7^	Ongoing
NCT00875654	Randomized, open-label, phase II	30	MSC	Autologous bone marrow	6 weeks	Intravenous	2×	No further details provided	Ongoing
NCT01714167	Non-randomized, open-label, phase I	30	MSC	Autologous bone marrow	3–6 months	Intraparenchymal	1×	2–4 × 10^6^	Ongoing
NCT01716481	Randomized, open-label, phase III	60	MSC	Autologous bone marrow	90 days	Intravenous	1×	No further details provided	Ongoing
NCT01962233	Open-label, phase I	10	MSC	Allogeneic cord blood	N/A	Intravenous	1×	1–8 × 10^8^, no further details provided	Ongoing
NCT01297413	Non-randomized, open-label, phase I/II	35	MSC	Allogeneic bone marrow	6 months	Intravenous	1×	0.5–1.5 × 10^6^	Ongoing
NCT02290483	Open-label, controlled, randomized, phase II	76	MNC	Autologous bone marrow	1–7 days	Intraarterial	1×	2 × 10^6^, 5 × 10^6^	Ongoing
NCT01468064	Double-blinded, randomized, controlled, phase II	90	Endothelial progenitor cells	Autologous bone marrow	5 weeks	Intravenous	2×	2.5 × 10^6^ per injection, 1 week interval	Ongoing
NCT01832428	Open-label, phase I/II	50	MNC	Autologous bone marrow	N/A	Intrathecal	3×	1 × 10^8^ per injection, 1 week interval	Ongoing
NCT01673932	Open-label, randomized, phase I	12	MNC	Allogeneic umbilical cord blood	6–60 months	Intraparenchymal	1×	10–40 × 10^6^	Ongoing
NCT01461720	Single-blinded, non-randomized, phase II	50	MSC	Autologous bone marrow	0.5–2 months	Intravenous	1×	N/A	Ongoing
NCT02378947	Double-blinded, randomized, phase I/II	18	MSC	Umbilical cord blood (allogeneic?)	0 day, 0 and 7 days	Intravenous	1×	2 × 10^8^ per injection, 1 week interval for the second injection	Ongoing
							2×	
NCT01501773	Open-label, randomized, phase II	120	MNC	Autologous bone marrow	7–30 days	Intravenous	1×	30–500 × 10^6^	Ongoing
NCT00950521	Open-label, randomized, phase II	30	CD34^+^	Autologous peripheral blood	6–60 months	Intraparenchymal	1×	2–8 × 10^6^	Ongoing
NCT00761982	Single-blinded, non-randomized, phase I/II	20	CD34^+^	Autologous bone marrow	5–9 days	Intraarterial	N/A	N/A	Ongoing
NCT00859014	Open-label, phase I	25	MNC	Autologous bone marrow	24–72 h	Intravenous	1×	10 × 10^6^/kg	Decreased hemoglobin (8 out of 25 patients), hypotension (1 out of 25 patients), musculoskeletal pain (9 out of 25 patients), hemorrhagic transformation of ischemic stroke (7 out of 25 patients)
NCT01028794	Open-label, non-randomized, phase I/II	12	MNC	Autologous bone marrow	7–10 days	Intravenous	1×	Cells derived from 25 or 50 ml bone marrow	Ongoing

## Complications Related to Intracerebral Cell Transplantation

The human brain is highly susceptible for damage emerging from surgical manipulation. Already minor structural defects can provoke tremendous functional consequences, so balancing safety and efficacy aspects of candidate cell delivery procedures is mandatory when translating an experimental treatment protocol into clinical application (67, 68). Therapies relying on cell populations for which a restorative potential is described would certainly benefit from cell deposition proximal to the lesion. It is currently unclear, whether this also holds true for the more widely anticipated bystander effects. Since these are believed to be exerted by growth factors and cytokines, it is reasonable to speculate that the therapeutic benefit may be increased when the cells are present in the lesion vicinity. Hence, local cell delivery is a viable option for clinical translation.

Intraparenchymal, stereotactically guided neurosurgical cell delivery allows the spatially precise deposition of therapeutic cells within or next to a lesion. It is also superior to other delivery approaches regarding absolute cell numbers reaching the brain (69). On the other hand, penetrating the cerebral parenchyma, e.g., by using a cannula, comes at the risk of inducing focal hemorrhage. For example, electrode deposition for deep brain stimulation in Parkinson’s disease was reported to cause such hemorrhages in about 3.1% of cases, with 1.4% being symptomatic (70). This risk is relatively low and often anticipated to be outweighed by the provided therapeutic benefit. Intracerebral cell transplantation has hence been chosen for a number of early stage clinical trials on stroke (45) and other neurodegenerative diseases (71, 72). However, the risk associated with an individual delivery may considerably accumulate when (i) multifocal cell deposits to address a larger or multifocal lesion(s) and/or (ii) repeated injections over a longer time course are required to ensure the therapeutic benefit (73). Indeed, complications related to the intraparenchymal cell implantation procedure have been observed in clinical studies, including headache, somnolence, and subdural hematoma (43, 46).

A possible alternative in such scenarios may be intraventricular/intrathecal cell delivery, which can be achieved by a single trajectory targeting lateral ventricles or by lumbar puncture and intrathecal injection. Administered cells are subsequently distributed along the ventricular walls, theoretically allowing a more widespread dissemination throughout the CNS than intraparenchymal approaches. Although intrathecal cell application methods have been predominantly used in experimental setups so far, some clinical case studies have been reported (65, 74). However, the rapid exchange of cerebrospinal fluid and its flow being generally directed toward the subarachnoid spaces including those in the spine render a targeted engraftment of intrathecally transplanted cells uncertain. Headache is a complication frequently reported after intrathecal cell transplantation (75). Intraventricular delivery further comes with a potential risk of hydrocephalus since the injected cells might adhere to the ventricular wall and cause dysfunction of cerebrospinal fluid circulation. Moreover, potential complications of intrathecal cell delivery, such as lumbosacral radiculopathies, causing pain and neurological deficits have been reported (66). Hence, thorough assessment of safety and efficacy profiles with respect to the desired mode of therapeutic action of a particular cell population is pivotal.

A recently investigated, interesting delivery option to the brain is intranasal cell administration (76). The proposed entry mechanism is an active, retrograde migration along fibers of the olfactory tract, which descent through the cribriform plate of the ethmoid bone. Intranasal cell administration is considered as a relatively safe way to target the rodent brain (77) and was found efficient to treat neonatal hypoxic–ischemic injuries (24, 78). It is less clear though whether the immunological barrier function of intranasal mucous tissue and the relatively long migrations distances in humans may compromise this option for clinical application. Intranasal delivery remains experimental for the time being, requiring more translational research on efficacy aspects, especially in species exhibiting larger brains.

## Adverse Events Following Systemic Intravascular Cell Administration

Systemic intravascular transplantation procedures comprise intravenous and intraarterial administration techniques. They are relatively easy to perform and less invasive than local cell delivery approaches. Therefore, intravascular application is believed to come with a generally favorable risk profile including for those patients being in a critical clinical condition. This is one of the main reasons why most current cell transplantation trials rely on systemic delivery techniques (79). Nevertheless, both intravenous and intraarterial cell transplantations are associated with a number of risks and complications. Those are evident in autologous as well as non-autologous transplantation paradigms and, importantly, are often underestimated due to the low level of invasiveness of systemic transplantation procedures.

These complications can also impair the transplant itself. For instance, non-autologous systemic stem cell transplantation can ignite an instant immune response against the cells (80) causing a loss of the graft or its functional impairment (this is discussed in detail below). An additional reduction of therapeutic cell numbers after systemic injection is caused by first passage cell loss in pulmonary capillaries, spleen, liver, and kidneys (81, 82). This trapping effect is believed to be the predominant cause for the overall low cell concentrations reaching the lesion area in systemic autologous transplantation settings. Cell size and diameter (see Table 1) are major determinants of vascular obstruction and complications emerging thereof (83). Smaller cell populations, such as bone marrow mononuclear cells (BM MNCs), are filtered less extensively than larger ones including neural stem cells (NSCs) or MSCs (84).

Pulmonary and splenic cell trapping may not be considered problematic at the first glance. First, pulmonary passage of larger cells can be increased by administration of strong vasodilators, such as nitroprusside (85) or nitric oxide directly, for which a beneficial effect on stroke has been described as well (86). Application of vasodilators would require continuous monitoring of blood pressure and in some cases methemoglobin, but would be justifiable in an intensive care setting. Second, some adult cell populations do not even need to enter the brain to exert a therapeutic benefit (87). However, a considerable fraction of transplanted cells usually becomes apoptotic before or during the transplantation procedure (88). Endogenous scavenger and clearance systems in the spleen remove blood-borne pathogens and apoptotic cells from circulation (89). Splenic clearance of circulating apoptotic cells helps to maintain self-tolerance and represents a central anti-inflammatory mechanism (90, 91), both of which can be considered beneficial after stroke (92). On the other hand, this system is also responsible for the clearance of neutrophils and its overload with excess amounts of transplanted cells could lead to increased levels of circulating neutrophils and deterioration of stroke outcome (88).

Microembolism is another important and detrimental complication, which has been reported after both intravenous and intraarterial cell delivery. Given its utmost relevance for systemic cell transplantation strategies in stroke, this complex complication is to be discussed in detail.

## Microembolism after Systemic Stem Cell Injection

Intravenous transplantation approaches often cause pulmonary cell microembolism, particularly when injecting larger cells. Embolus formation from transplanted MSCs in the lungs of small (93) and large animals (94) has been described for more than a decade. Originally being discussed as an undesired side effect, primarily compromising the number of therapeutic cells being available at the therapeutic target, pulmonary microemboli have been increasingly recognized as an important safety concern. In their seminal study, Lee and colleagues showed that intravenous delivery of commonly used therapeutic MSC doses (2 × 10^6^ per animal) was lethal in 10% of transplanted mice (95). Higher MSC doses (3 × 10^6^ per subject) caused 80% fatalities from severe pulmonary dysfunction within minutes with the effect mainly being attributed to the PODXL^low^/CD49f^low^ MSC subfraction that represents matured progenitors (95). Surface integrin expression and consequently the risk for pulmonary entrapment further depends on the donor age (82), which might require an individual risk assessment in autologous cell therapies. Importantly, it has been shown that the number of MSCs clotting pulmonary capillaries is not effectively diminished when infusing lower MSC concentrations or after blocking surface integrins (96).

Targeted intraarterial cell delivery, for example, into the internal carotids or the M1 branch of the middle cerebral artery, is often discussed as viable alternative circumventing potential drawbacks of intravenous cell administration. The approach is further believed to deliver significantly larger amounts of cells to the desired location (97). Nevertheless, microembolism upon intraarterial cell delivery has been reported in accordance to the situation observed in the venous branch. The vessel blockage can occur already at the precapillary level, resulting in a massive and immediate blood flow drop in the arteriole and downstream capillaries (98). This local hypoxic–ischemic environment is believed not only to cause a loss of around 85% of transplanted cells in the long run but also to provide additional hypoxic–ischemic stress to the surrounding areas. This is obviously detrimental in tissues with a high-oxygen consumption and nutritional demand, such as the myocardium. It can ultimately result in additional, macroscopic ischemic myocardial damage (99). Of note, the administration of stem cell populations, such as MSC, can further trigger the procoagulatory cascade via surface expression of tissue factor (100). This in turn causes thromboembolism aggravating mechanical vascular obstruction and closing the loop to immunological complications of intravascular stem cell applications, which are discussed below.

In accordance to the situation in the heart, the induction of focal cerebral ischemic injury has been reported after intraarterial MSC injection to the brain. Cui and coworkers showed immediate, sometimes transient cerebral blood flow (CBF) decreases after injection of allogeneic BM-derived MSC into rats with frequency and extent correlating to the number of infused cells (25). The CBF drops were extensive enough to cause cerebral microinfarcts as determined by magnetic resonance imaging and histology when 0.5 × 10^6^ or more cells were administrated. Infusion of higher cell numbers also tended to coincide with reduced functional performance, but the results were not statistically significant apart from a clearly compromised locomotor activity. This was potentially due to the variable lesion pattern caused by intraarterial MSC injection and the resulting heterogeneous functional impairment, both requiring very large samples sizes to reach adequate statistical power. On the other hand, injection of 0.1–0.25 × 10^6^ cells was reported safe (25, 101).

A cell diameter of around 15–16 μm, resulting in volumes between approximately 1750 and 2150 μm^3^ (if simplistically considering the cell body to resemble a sphere), appears as a critical threshold for the occurrence of intravascular embolism (84). This diameter is, for example, observed in BM-derived multipotent adult progenitor cells (MAPCs), whereas most MSC population are significantly larger (18 μm and more). This assumption is corroborated by results from a recent phase IIb clinical trial on MAPCs by Athersys Inc., enrolling 65 patients to the cell treatment group. Although some evidence for limited pulmonary trapping was observed preclinically (84), intravenous MAPC injection (up to 1.2 × 10^9^ cells per patient) did not increase, but even reduced the frequency of pulmonary complications (mainly pneumonia) in treated patients (see Table 2). One may assume that the therapeutic benefits have outweighed potential complications of pulmonary MAPC trapping in this scenario.

Recent evidence indicates that infusion velocity and the type of administered cells also play a role in cell-prone vascular occlusion, particularly after intraarterial administration. In rats, low-infusion speeds (<0.1 ml/min) seem to facilitate cell clotting and lead to a higher incidence of vascular obstruction. Surprisingly, relatively high-infusion speeds (0.3 to >1 ml/min) also result in secondary ischemic lesions for so far unclear reasons. Infusion velocities around 0.17–0.2 ml/min resulted in best safety profiles in Sprague-Dawley and Wistar rats, even allowing administration of up to 1 × 10^6^ MSCs without safety restriction (25, 102). Interestingly, there was no safety profile difference between application techniques targeting the external or common carotid artery, but this does not necessarily translate to human patients (102). When cells of similar diameters, i.e., 15 μm, are transplanted, some authors report intracapillary trapping and obstruction [Ref. (84) for NSCs, MSCs and, less pronounced, for MAPCs], whereas others do not [Ref. (102) for glial-restricted precursor cells, GRPs]. The precise reasons behind this still have to be elucidated, but differences in soma rigidity might provide a potential explanation. Early stem and progenitor, and also tumor cells present a less rigid cytoskeleton and increased deformability (103, 104), which may in turn facilitate capillary transition.

Safety of intravascular cell infusion seems to be a complex function determined by the targeted circulation compartment, cell type, size, and infusion speed. A few clinical cases of cell-borne embolism have been reported, although not in stroke patients yet. Diffusion weighted MRI revealed small, focal, and asymptomatic lesions 1 day after intraarterial injection of MSCs to patients suffering from multiple system atrophy (92). Pulmonary embolism has also been described after intravenous infusion of adipose tissue-derived stem cells (105) and is even speculated to have caused a fatality (100) in a recent clinical study. This clearly calls for additional safety investigations including both clinical and animal studies. It should be realized that preclinical research on safety aspects is complicated even further by significant interspecies differences in vessel diameter, rheology, coagulation, and platelet function, potentially preventing a direct transfer from rodent data to human subjects. Large animal models (106–108) might provide some benefits in the translational process, but a careful consideration of human anatomy and stroke pathophysiology remains essential when designing clinical studies.

## Neoplasms, Tissue Overgrowth, and Ectopic Cell Engraftment

The generation of functional brain tissue and particularly of the human neocortex during embryo- and fetogenesis is a complex and very well controlled process (109). Although our knowledge about its precise orchestration remains limited (110), it is well known that the process is highly susceptible to even minor external disturbances (111). On the other hand, there is a strict spatial and temporal limitation of structured postnatal neurogenesis (112). The intracerebral transplantation of multi- and pluripotent stem cell populations in an attempt to restore the lesioned brain, but without providing the biochemical and anatomical environment required for structured neuro- and gliogenesis therefore comes at the risk of uncontrolled proliferation emerging from the graft. The landmark paper by Erdö and coworkers describes for the first time the generation of aggressively growing neoplastic formations resembling primitive neural structures after stereotactic transplantation of allogeneic embryonic stem cells (ESCs) into the mouse brain (12). The initial enthusiasm fueled by positive results from experimental cell therapies for stroke was further damped when teratoma and, occasionally, teratocarcinoma formation were reported for many other pluri- and multipotent cell populations including induced pluripotent stem (iPS) cells (36). The phenomenon has been observed in numerous mammalian species and in both allo- and xenograft paradigms. Teratoma formation is thought to originate from two distinct preconditions (15). It first requires the presence of extensively mitotic, insufficiently linage-committed cells within the transplant. Moreover, the post-ischemic environment itself is thought to provide a ground for teratoma formation due to the strong expression of antiapoptotic messengers (113) as well as proliferation-promoting cytokines including basic fibroblast growth factor (114), brain-derived neurotrophic factor (115), nerve growth factor (116), and vascular endothelial growth factor (117). Teratoma formation has not been reported following the transplantation of highly migratory, lineage-restricted GRPs so far.

Another important safety concern particularly after intracerebral cell transplantation is extensive growth from the graft at the expense of the local host tissue, but without generation of germ cell tumors. In this scenario, a continuous proliferation of mitotic neural progenitor cells results in the extensive generation of immature neurons, which excludes the neuropathological classification of such tumors as teratomas. Importantly, this phenomenon is independent from the local, growth-permissive post-ischemic environment. It rather seems to emerge from extensive intrinsic proliferation activity within the graft (15) and may resemble a proliferation peak as physiologically observed during mammalian CNS development (118). Pathophysiological sequelae of graft overgrowth include compression of local brain tissue and capillaries leading to extensive multifocal necrosis. Strategies to prevent the formation of neoplastic structures comprise the application of thorough cell selection protocols (119) and neuronal predifferentiation toward immature, but postmitotic cells (120) prior to transplantation. A more recent approach is the application of transdifferentiation technologies, which allow the direct creation of neurons from somatic cells (121).

Uncontrolled growth from transplanted cells is not restricted to the side of graft implantation. It has been shown that therapeutic stem cell populations exhibit an expressive migration capacity, which allows them to reach ischemic foci even if located in the contralateral hemisphere (16, 122). Under so far undetermined conditions, transplanted cells can also utilize their migration capabilities to repopulate unlesioned, remote locations. Extensive migration of locally administered NSCs along the spinal cord and within the CSF space was shown to result in numerous ectopic engraftments containing proliferating cells as well as postmitotic neurons and glia across the CNS (123). The biological and functional consequences of such ectopic colonies are currently unclear. Nevertheless, they represent a safety concern since uncontrolled, ectopic proliferation, comes at the risk for tissue overgrowth including disseminated host tissue compression, or potentially even more aggressive tumor formation.

Ectopic survival and proliferation of systemically engrafted stem cells has been described occasionally. For example, allogeneic and autologous MSCs have been reported to survive following intravenous administration and sublethal irradiation in a baboon model. Cells have been identified in the gut, kidney, thymus, liver, and skin after up to 21 months (124), but neoplasm formation was not reported. A clinical case reported donor-derived glioneuronal neoplasm after intracerebellar and intrathecal injection of human fetal NSCs in a commercial stem cell transplantation center not meeting Western standards (64). The risk of such tumor formation is most likely reduced or even absent when using adult stem cells and particularly allogeneic populations as well as systemic delivery since the majority of them will be eventually rejected by the host’s immune system. However, long-term culture can cause chromosomal alteration (125, 126) and clonal growth in neonatal and adult stem cells (127), so the potential induction of neoplasms from an adult cell graft has to be monitored carefully. Moreover, donor cell-derived leukemia has been reported after systemic transplantation of allogeneic hematopoietic stem cell (128) and umbilical cord blood (129) in some patients with hematological diseases. The particular reasons for this rare phenomenon remain for further investigation and it is unclear whether this is of relevance for cell transplantation approaches after stroke.

Interestingly, ectopic cell engraftment has also been described following intranasal delivery. Luciferase-expressing (F3-effluc-positive) immortalized human NSCs were intranasally administered to immunocompromised BALB/c nude mice. Serial bioluminescence imaging (BLI) was conducted to monitor cerebral engraftment non-invasively (130). BLI signals were obtained from the brain of about one-third of transplanted animals, but concomitantly observed in the lungs of about 45% of all cell-treated mice. Despite a transient disappearance, strong bioluminescence signals were found emerging from the lungs 2 weeks after transplantation. Post-mortem histopathological investigation revealed that the signals originated from F3-effluc-positive pulmonary tumors. Nevertheless, such severe complications can be considered unlikely in immunocompetent recipients given the immunological barrier function of the airway mucous tissue after intranasal delivery, as well as instant, blood-mediated inflammatory reactions (131) or other mechanisms of systemic graft rejection, and, finally, continuous immunosurveillance and adaptive immune responses (132) in most tissues. It therefore seems logical that very few papers report a highly limited ectopic engraftment and long-term survival outside the CNS after transplantation into immunocompetent hosts (133). However, should cell therapies become a routine procedure in the future, they will likely rely on allogeneic transplants for logistic reasons and may therefore require concomitant immunosuppression, which, together with the commonly observed post-stroke immunodepression (134), may theoretically open a small window for such ectopic engraftment to materialize.

## Immunological Consequences of Cell Therapies

The transplantation of foreign cells, namely, from allogeneic or xenogeneic sources can induce the two prototypes of immune responses: graft rejection and graft-versus-host disease (GvHD). Both occur because of major histocompatibility complex molecule (MHC) mismatches between transplanted and endogenous cells. Graft rejection is induced by circulating antibodies and T cells recognizing foreign MHC molecules (135). The risk for immunological rejection can be reduced either by matching the MHC types as clinically practiced in allogeneic hematopoietic stem cell transplantation, by immunosuppressive treatment, or by using cells with low expression of MHC-I molecules (i.e., low immunogenicity) as it is described for several stem cell populations (136, 137). However, the latter advantage could be limited by recipient natural killer (NK) cells that detect and kill cells without MHC-I expression (138). It is still controversially discussed whether MHC-I^low^ stem cells are protected from NK cell lysis by concurrent low expression of co-stimulatory molecules, such as NKG2D ligands (139, 140). However, when cells graft within an inflammatory environment, such as the ischemic brain tissue, they may upregulate MHC-I (136, 141, 142) and thus secondarily increase their risk for rejection (140). Even though allogeneic and syngeneic cells exhibit the same MHC molecules as the recipient, they are not entirely protected from being rejected. Genetic engineering to express therapeutically relevant proteins, function-determining transcription factors or reporter molecules (143, 144) may increase immunogenicity and the risk for rejection. This aspect is especially relevant for the emerging field of iPS cells (145, 146) and may require careful screening of cell lots prior to clinical use (147). Graft rejection generally implies a reduction or even a complete loss of therapeutic effects, either by a loss of transplanted cells (139, 148) or their functional impairment (149). To the best of our knowledge, it is yet unknown whether the immunological process of cell rejection itself may additionally harm recipient tissue, especially after intracerebral transplantation with relatively large cell depots.

In contrast to graft rejection, GvHD only occurs when transplanted MHC-mismatched cells contain leukocytes or cells with leukocyte function. Symptoms of GvHD not only concern primarily the mucosa, skin, and liver (150) but may also affect the brain (151). In the stroke field, this is solely relevant for umbilical cord blood cell transplantation (152, 153), since BM MNCs are mainly used autologously (51, 61).

One of the most frequently used cell type in stroke studies, MSC, exhibit strong immunomodulatory properties that are even used to treat therapy-resistant GvHD (154, 155). The mechanisms of MSC-induced immunosuppression are not completely understood, but rely on the expression of anti-inflammatory mediators, such as IL-10, TGF-β, and prostaglandin E2, and of co-stimulatory molecules, such as programed death ligands and Fas ligand [reviewed by Ma et al. (156)]. These immunosuppressive properties seem to be one important mechanism for the therapeutic efficacy of MSCs (9). In contrast, MSC-induced systemic immunosuppression could also be extremely harmful after stroke by amplifying the post-stroke immune deficiency syndrome and hence the risk for infections (134, 157). This hypothesis was not confirmed by a recent mouse study (158), but was discussed as reason for early death in a clinical GvHD trial (157). Further investigations should address that issue to ensure safety in clinical MSC transplantation after stroke.

One important advantage of cell therapies over one-target pharmacological approaches is the capacity of certain cells to home toward the injured tissue and to simultaneously manipulate a variety of pathophysiological processes. However, this is a positivistic view, since the interaction between the cell graft and the inflammatory niche is far from being understood. For example, it has been described that MSCs and NSCs express toll-like receptors (TLRs) and that TLR-signaling affects important cell functions including differentiation, proliferation, and migration (159–162). However, after stroke, the ischemic tissue is flooded with damage-associated molecular patterns (DAMPs), which activate several TLRs and drive post-stroke inflammation (163). *In vitro* studies revealed that the activation of the TLR4 pathway causes an increased secretion of pro-inflammatory mediators both by MSCs (164, 165) and NSCs (166). The sole co-cultivation of MSCs with macrophages also induced a pro-inflammatory MSC phenotype secreting large amounts of IL-6 and different chemotactic cytokines that could attract leukocytes (167). Consequently, it is plausible that transplanted cells, which reach the ischemic brain, could further amplify detrimental inflammation and thus contribute to brain damage. A better understanding of the impact of the microenvironment on the function of transplanted cells is necessary to dissect harmful and beneficial immune effects of transplanted cells.

Recent evidence indicates that stroke is significantly determined by thromboinflammatory mechanisms. For instance, regulatory T cells strongly interact with platelets and activated brain endothelial cells to form microvascular thrombosis in the acute stage of stroke. Ablation of regulatory T cells, however, successfully restored CBF and ameliorate functional outcome (168). It is imaginable that the transplantation of cells with strong homing and transmigration capabilities may also support thromboinflammation and thus contribute to brain damage. In fact, live imaging of MSCs homing toward inflammatory foci revealed that almost 50% of intravenously injected MSCs form intravascular clusters with platelets and neutrophils at the site of inflammation (169). Activation of TLR pathways further causes an upregulation of VCAM-1 and ICAM-1 on the surface of MSCs (164). Adhering and transmigrating MSCs at the ischemic brain endothelium may thus act as toeholds for adjacent leukocytes and exacerbate thromboinflammation. Although the literature indicates that seemingly few intravenously transplanted cells reach the ischemic brain as discussed above, this potential adverse mechanism should be kept in mind when cells were engineered to improve homing and transmigration.

## Seizures

Given the potential complications of seizures, they are among the more severe adverse events to expect. Moreover, seizures represent a safety concern since they must be controlled by antiepileptics, although such medication can impair the recovery process following stroke (170). The post-ischemic brain is susceptible to various stimuli potentially inducing seizures. For instance, it is well known from animal studies that the excitability of perilesional cortical neurons is increased because of altered glutamate and GABA signaling (171, 172). In line with this, early seizures are observed in 2–9% of patients after stroke (173, 174). Interestingly, a recent study reported that two patients out of seven after intraarterial administration and all patients (five out of five) after intravenous administration of autologous BM MNCs suffered seizures (57). This is far above the seizure frequency that one would expect to occur spontaneously. Partial or generalized seizures were also observed in other clinical studies (43, 49, 53). A potential causal link between cell transplantation and increased seizure frequency has not been established, but is known that seizures represent a relatively common complication of cell treatments for non-neurological conditions (175). Seizures accounted for about half of the CNS-related complications of hematopoietic stem cell therapy in pediatric patients suffering from hematological malignancies (176), but it is challenging to clearly discriminate cause and effect from these observations. Establishing a causal relationship between increased seizure frequency and therapy attempts using adult stem cell populations following stroke is also impaired by the small number of patients enrolled to these early phase trials studies, some of which are non-randomized, uncontrolled phase I studies.

It is easier to speculate about a potential pathomechanism leading to higher seizure frequencies after administration of cells with neurogenic properties. Neurons emerging from ectopic stem cell colonies were shown to send axons into the host CNS after local transplantation to a site of severe traumatic spinal cord injury (123). It could be speculated that local circuit integration may pave the way for seizures, which could also become evident in stroke, although a physiological integration of graft-derived neurons is principally possible upon stroke (14). When transplanting less potent, adult cells populations or relying on systemic administration, seizures may be a sequel of cerebral microembolism (intraarterial approach) or potentially systemic immune responses. Of note, virtually no preclinical research report is available reporting on seizures as a potential adverse event in animals receiving cell therapy in stroke, potentially due to the difficulties to detect other than generalized seizures in laboratory rodents. Another factor potentially contributing to this is the relatively short post-stroke surveillance times (between 2 weeks and 1 month), which may be too short to observe seizures, at least those arising from disorganized neuronal cell engraftment. Finally, the stroke model used may play a role as well. Post-stroke epileptogenesis has been observed in the Rose bengal photothrombosis model, but not in the filament model (177).

## The Role of Common Comorbidities and Drug–Cell Interactions

Current knowledge suggests that the translational failure in the development of novel treatment strategies for stroke is partly due to the inability of most animal models to adequately reflect the complex pathophysiological situation in humans (178). This particularly accounts for factors including age and polypharmacology as well as for comorbidities, such as hypertension and diabetes (179). The presence of such factors is believed to have impaired the therapeutic efficacy of virtually all neuroprotective drug candidates developed in the past. The currently emerging field of cell therapies might therefore face similar challenges.

Diabetes represents one of the most important and most frequent diseases leading to cerebrovascular disorders in Western societies, but experimental studies in diabetic subjects are relatively rare. Chen and coworkers investigated the effect of bone marrow-derived MSCs, intravenously administered 24 h after a 2 h transient middle cerebral artery occlusion in streptozotocin-induced type 1 diabetes rats (180). The applied cell transplantation protocol was repeatedly shown to beneficially influence stroke outcome by this and other groups. However, MSC therapy surprisingly increased the mortality without affecting lesion volume or functional outcome in diabetic rats. This was explained by an increased BBB opening leading to a higher incidence of hemorrhagic transformation. MSC treatment also induced narrowing of cerebral arterioles (up to the level of vascular occlusion), neointima formation in the internal carotid artery, and further facilitated atherosclerotic transformation of cerebral vessel walls. The postulated cause was an increased expression of angiogenin in the treatment group, which can aggravate microvascular pathologies particularly in diabetics (181). These findings indicate that the importance of comorbidities not only as an efficacy-limiting factor for cell therapies in stroke but also as being highly important for safety considerations.

Another interesting aspect is the possibility of interaction between a pharmaceutical drug and administered therapeutic cells. Given the wide spectrum of medication, a stroke patient typically receives before and after the event, it comes as a surprise that beneficial or detrimental interaction has only been rarely investigated so far. Most of the data being available on drug–cell interactions are not primarily focused to discover such interplays, but to tests whether or not the combination treatment exerts an additional benefit. For instance, during the investigation of such combination therapy, a potential detrimental interaction between granulocyte colony-stimulating factor (G-CSF) and BM MNCs has been discovered (88). This was based on the unfortunate timing of the BM MNCs injection, falling together with the moderate, G-CSF-induced granulocyte peak around 48 h after stroke. Blocking the splenic scavenger system for apoptotic cells by administered BM MNCs as discussed above prevented the removal of apoptotic granulocytes from the circulation, providing a systemic pro-inflammatory bias increasing the numbers of granulocytes in the lesioned hemisphere. This ultimately abolished the beneficial effects provided by G-CSF. The combination was also inefficient in a study using normotensive, but senescent animals (182) as well as after replacing BM MNC with MSCs (183).

Apart from potential detrimental effects exerted by cell therapies, the pathophysiological environment in turn can compromise the efficacy of administered cells. This is especially relevant since stroke is a disease of the elderly population, in which endogenous recovery processes and therapeutic interventions are likely to be impaired by comorbidities and co-existing risk factors (184). A prominent aspect is a subtle pro-inflammatory status of the aging brain, making it susceptible to ischemic damage and both accelerating and aggravating post-stroke neuroinflammation. This situation severely limits therapeutic efficacy of cell treatments and should be considered in preclinical research (185). Moreover, the complex environment in a comorbid patient population may trigger unintended or detrimental responses that have not been observed to that extend in animal models. Implications of this situation for the translational process, in principle applying to all forms of experimental therapies, have recently been reviewed by Hermann and Chopp (186). In particular, three highly relevant scenarios have been pointed out: an effect being beneficial in an otherwise healthy model organism may (i) provoke an ambiguous response or (ii) be even detrimental in the pathophysiological context, and (iii) the desired therapeutic effect could interfere with the response to another treatment (e.g., parallel medication). For example, a recent study revealed such interference between MSCs and dexamethasone when conjointly applied as an anti-inflammatory treatment (187). Here, the MSC-induced reduction of T cell proliferation was reversed in a dose-dependent manner by dexamethasone, which also reversed the positive impact of MSC transplantation in an animal model of liver fibrosis. Moreover, some drug classes, such as neuroleptics, have been shown to impair post-stroke recovery (188) and may therefore also interfere with cell-based effects on functional recovery. On the other side, a thorough investigation of drug–cell interactions can pave the way to therapeutic utilization of potential synergistic effects, providing an add-on benefit as exemplified by some recent investigations (189, 190). Since the field is both highly complex and severely underinvestigated particularly with respect to those drug classes commonly given to stroke patients (e.g., antihypertensive, antidiabetics, thrombolytics, and anticoagulants), further preclinical research on these aspects is highly necessary.

## Problems Related to Improper Cell Product Manufacturing

Application of therapeutic cells in the clinical environment demands cell production under conditions of Good Manufacturing Practice (GMP). Moreover, therapeutic cell products are subject to the advanced therapy medicinal cell product (ATMP) legal framework of the European Medicines Agency (EMA) or similar regulations, such as supervised by the Office of Cellular, Tissue and Gene Therapies of the Food and Drug Administration (FDA) in the United States. The strict regulatory control of therapeutic cell production is intended to enhance safety of novel cell products for patients by excluding the presence of viral contaminants, mycoplasma, endotoxins, or xenogeneic supplements. This makes adverse events related to the manufacturing process very unlikely. Nevertheless, some countries have issued less strict regulations, which may increase the risk for manufacturing-related complications. Indeed, some adverse events reported after cell therapy are related to improper cell product manufacturing. For example, dimethylsulfoxide (DMSO), a cytotoxic cryoprotectant frequently used in protocols for laboratory- and GMP-grade cryopreservation of cells, is occasionally found in the cell suspension. DMSO has been reported to cause allergy reactions (191), while aseptic meningitis has been reported after intrathecal administration of DMSO-containing suspensions (75). More severe complications like transient encephalopathy, stroke, and myocardial infarction have also been reported, which might be due to the vasospasm effect of DMSO after systemic infusion (192). Complications related to cell production or the application of unsafe cellular preparations represents a severe safety concern in destinations of stem cell tourism (193). The highly inappropriate systematic investigation and follow-up of these cases by dubious private stem cell therapy providers subjects any quantitative statement or risk estimation to speculation, but it is unfortunately undoubted that such cases exist. An efficient way to prevent these is public education (194). This is also important since commercial exploitation and causing harm to patients being desperately in seek for a cure will for sure compromise the scientific efforts of serious stem cell researchers and clinicians and may even bring down the entire field.

## Conclusion and Recommendations

Each therapy causes side effects and adverse events. Hence, it does not come as a surprise that complications have also been reported for cell therapies upon stroke. Although a final conclusive statement would clearly be premature, the risk for severe adverse events in human patients seems to be relatively low considering the data derived from clinical trials that have been conducted so far. Even if present more frequently, side effects would not generally comprise the value provided by cell therapies in stroke as long as they are controllable and outweighed by the benefit provided to patients. Importantly, a number of severe and potentially devastating complications including secondary brain infarction after intraarterial cell delivery and teratoma formation following transplantation of more naïve stem cell populations have been detected by carefully conducted preclinical safety studies. This has restricted the incidence of severe complications to a small number of patients, many of whom have fallen victim to dubious commercial cell transplantation services. On the other side, a better understanding of potential risks will facilitate the translational process for stem cell therapies in stroke and will make this translation safer by providing the community with a reasonable chance to work out protocols circumventing such complications. A sound awareness of potential side effects and limitations of cell therapies for stroke may even inform new concepts about how these therapies can be further amended regarding their efficacy or at least help to define the particular conditions, which come with the highest chances to see therapeutic success. Weighting these aspects, we recommend considering the following points during translational stem cell research in stroke:

- The STAIR and STEPS expert community guidelines should be consulted and followed to maximize the value of research projects.
- Non-invasive monitoring techniques and detailed post-mortem histological investigations should be applied whenever possible to reduce the risk of leaving potential complications of stem cell therapies undetected in preclinical experiments.
- Special attention should be given on potentially altered effects of cell therapies in the pathophysiological context, presence of comorbidities, and an aged population.
- Multicenter preclinical investigations could help to target complex pathological conditions (e.g., polypharmacy, comorbidities) under which potential side effects may materialize. Efficacy of a particular approach should be confirmed before moving on to clinical investigations.
- Careful safety assessments are particularly warranted for experimental tandem treatments reported in preclinical literature, for example, combining cell transplantation with pharmacological therapies.
- Large animal experiments help to define optimal routes of cell administration, the exploration of novel, imaging-based clinical monitoring protocols, and should be considered for long-term safety assessments.
- Potential systemic effects (e.g., immunological responses, ectopic cell engraftment, and proliferation) must be taken into consideration when assessing system effects of both local and systemic cell applications.
- During the design of a clinical trial, the human anatomy (patho-)physiology should be considered when interpreting results from preclinical studies and deriving implications thereof.
- Adverse events should be carefully documented and reported, even if considered non-study related or indifferent between treatment and control groups.
- Clinical trials should be planned in such a way that they utilize available non-invasive monitoring techniques to the optimal benefit as well as frequent and long enough to ensure the detection of potential rare or late complications.

In light of the concerns and complications discussed in this review as well as the recommendations listed above, more preclinical and, later on, clinical safety studies should be conducted to maximize the potential benefits of cell therapies for stroke patients while reducing related risks. Moreover, we have to exploit all option to minimize the possibility of leaving potential complications undetected particularly in early stage clinical trials. A conservative study design focusing on additional safety rather than accessory efficacy endpoints appears imperative in this context.

## Author Contributions

JB drafted the manuscript idea, designed its structure, and coordinated the writing of this manuscript; JB also contributed the majority of paragraphs. AA has performed online research on tables 1 and 2 and made thorough revisions of the entire manuscript text. PW has contributed to manuscript writing and design of figure 1. JJ contributed to manuscript writing and research on table content. LC performed research on tables 1 and 2, contributed to all paragraphs despite introduction and performed thorough revisions on the entire manuscript. DW contributed significantly to the design of the manuscript, writing and table design. DW also conducted thorough revisions to the entire manuscript. All authors have reviewed the final manuscript version and agreed on its content.

## Conflict of Interest Statement

The authors declare neither financial nor other (e.g., intellectual property) conflict of interest. Only intramural funds were used for the work on this manuscript.
